# Biomimetic Design of a New Semi-Rigid Spatial Mesh Antenna Reflector

**DOI:** 10.3390/biomimetics9020074

**Published:** 2024-01-25

**Authors:** Hualong Xie, Yuqing Feng, Qunfeng Bi, Xiaofei Ma, Junfeng Zhao

**Affiliations:** 1 School of Mechanical Engineering and Automation, Northeastern University, Shenyang 110819, China; 2 Xi’an Branch, Chinese Academy of Space Technology, Xi’an 710100, China; fengyq0216@sina.com (Y.F.);

**Keywords:** bionics, space mesh antenna, reticular reflective surface, reflective surface accuracy

## Abstract

The reflective surface accuracy (RSA) of traditional space mesh antennas typically ranges from 0.2 to 6 mmRMS. To improve the RSA, an active control scheme can be employed, although it presents challenges in determining the installation position of the actuator. In this study, we propose a novel design for a semi-rigid cable mesh that combines rigid members and a flexible woven mesh, drawing inspiration from both rigid ribbed antennas and biomimicry. Initially, we investigate the planar mesh topology of spider webs and determine the bionic cable surface’s mesh topology based on the existing hexagonal meshing method, with RSA serving as the evaluation criterion. Subsequently, through motion simulations and careful observation, we establish the offset angle as the key design parameter for the bionic mesh and complete the design of the bionic cable mesh accordingly. Finally, by analyzing the impact of the node quantity on RSA, we determine a layout scheme for the flexible woven mesh with a variable number of nodes, ultimately settling for 26 nodes. Our results demonstrate that the inclusion of numerous rigid components on the bionic cable mesh surface offers viable installation positions for the actuator of the space mesh antenna. The reflector accuracy achieved is 0.196 mmRMS, slightly surpassing the lower limit of reflector accuracy observed in most traditional space-space mesh antennas. This design presents a fresh research perspective on combining active control schemes with reflective surfaces, offering the potential to enhance the RSA of traditional rigid rib antennas to a certain extent.

## 1. Introduction

**Figure 1:**
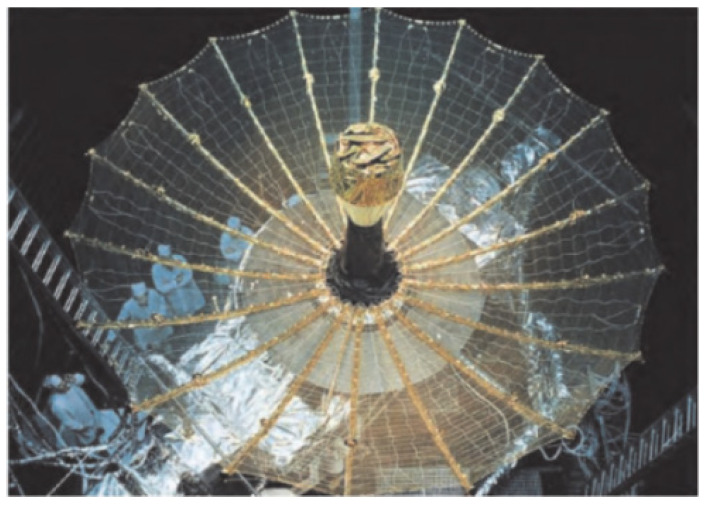
The American TDRS relay satellite is equipped with a radial ribbed antenna.

Space antennas are primarily installed on satellites, spacecraft, and other spaceborne platforms [1], facilitating two-way information transmission between these vehicles and ground stations. Operating as the “ears” or “loudspeakers” of spacecraft [2,3], space antennas have become an indispensable and vital component, influencing and constraining the performance and functionality of entire wireless communication systems and, by extension, the spacecraft itself. Deployable space antennas utilized in spacecraft can generally be categorized into four types: mesh deployable antennas, solid surface deployable antennas, inflatable deployable antennas, and semi-rigid deployable antennas [4,5]. Among these, the mesh deployable antenna, commonly known as the mesh antenna, stands out as the predominant focus of both domestic and international research and application efforts. Space mesh antennas can be further classified into ring truss type [6,7,8,9,10], center radial rib type as shown in Figure 1 [11], frame type [12,13], one-dimensional extension cylinder type [14], etc. [15,16], based on the variations in metal mesh surface expansion and supporting structures. Guangda Ding proposed a method for linear phased array optimization synthesis in the presence of calibration error and cross-coupling [17]. Qunbiao Wang presents a novel interval analysis method based on an ad hoc auxiliary formulation to effectively manage the influence of deployment angle error [18]. Shaopeng Pan proposes a new type of antenna design scheme that can be used for IoT relay communication [19].

**Figure 2:**
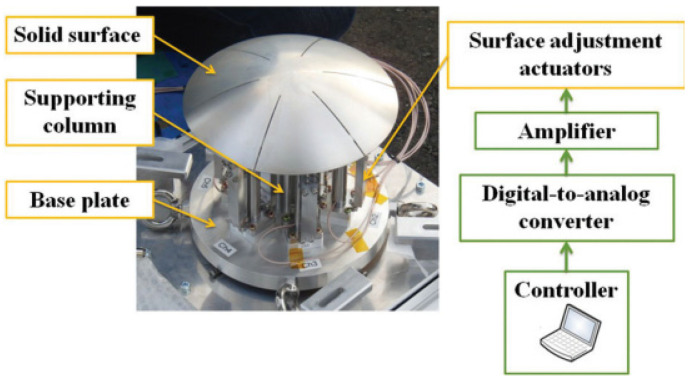
High-accuracy antenna system equipped with a smart reconfigurable reflector.

The reflective surface accuracy (RSA) of space mesh antennas significantly impacts their electrical performance. Currently, active control schemes are frequently employed to adjust RSA for different frequencies. Variations exist in the active control schemes adopted by different antenna types. For instance, certain solid reflective surface antennas employ vertical actuation [20] as shown in Figure 2, delivering a favorable actuation effect but increasing the antenna’s overall mass. In-plane actuation mode, utilized by some solid reflective surface antennas, balances actuation effects with antenna mass [21]. Cable net reflective surface antennas may be installed on cables [22], yet effective installation methods remain an open challenge, and such antennas are susceptible to wire entanglement during the folding process. Shell film reflective surface antennas predominantly employ the direct control schemes of the reflective surface [23,24]. In summary, the current research and the application of active control schemes for reflective surfaces face notable challenges, particularly regarding the actuator’s installation position. The cable mesh design of new space mesh antennas should address the need for additional actuator installation locations.

Zhen Chen proposes a compressive channel estimation technique for IRS-assisted mmWave multi-input and multi-output (MIMO) system, and a hybrid multiobjective evolutionary paradigm is developed to solve the sparse recovery problem, which can overcome the difficulty in the choice of regularization parameter value [25]. Shengchuan Jiang proposes a three-stage practical and economical layout planning approach for UWB base stations, including determining the deployment strategy and layout parameters as well as the comprehensive adjustment and the scheme verification [26]. Guoqing Zhou designs a LiDAR echo signal detection timing control system. The system combines PMT adjustable voltage control gain technology and applies the weak underwater echo signal that is amplified to facilitate the subsequent collection [27]. Yong Zhang proposed to produce an on-chip Luneburg lens by integrating gradient metamaterial structures and silicon waveguides [28]. Kwok L. Chung proposed a novel technique of consolidating microstrip lines (MLs) and coplanar waveguide (CPW) lines on a single dielectric substrate, designated as the composite microstrip/CPW line [29]. Zhimin An proposed a new multispectral stealth metastructure, which can achieve high temperature microwave absorption and infrared stealth simultaneously. This research provides an effective way to design microwave infrared compatible stealthy materials with huge multifunction [30]. Yu Yao proposed that the anti-eavesdropping scheme in CAVs networks is developed through the use of cognitive risk control (CRC)-based vehicular joint radar-communication (JRC) system. Numerical experiments have presented that the developed approach has an anticipated performance in terms of some risk assessment indicators [31].

For addressing the integration challenge between the existing spatial mesh antenna structures and the active control schemes, this study is based on biomimicry. It leverages the traditional rigid ribbed space mesh antenna as the structural foundation, drawing inspiration from spider web plane topology to propose a novel cable mesh surface design scheme incorporating rigid members and flexible woven mesh composites. Concurrently, the RSA of this new cable mesh surface is analyzed.

## 2. Analysis of Spider Web Characteristics and Bionic Evolution

The reflective surface of a space mesh antenna resembles the planar configuration of a spider web, showcasing its robust stability. Consequently, this study adopts the spider web as a bionic model for investigation, culminating in the design of a novel cable mesh surface through an analysis of the spider web’s planar structure in conjunction with the characteristics of space antennas.

**Figure 3:**
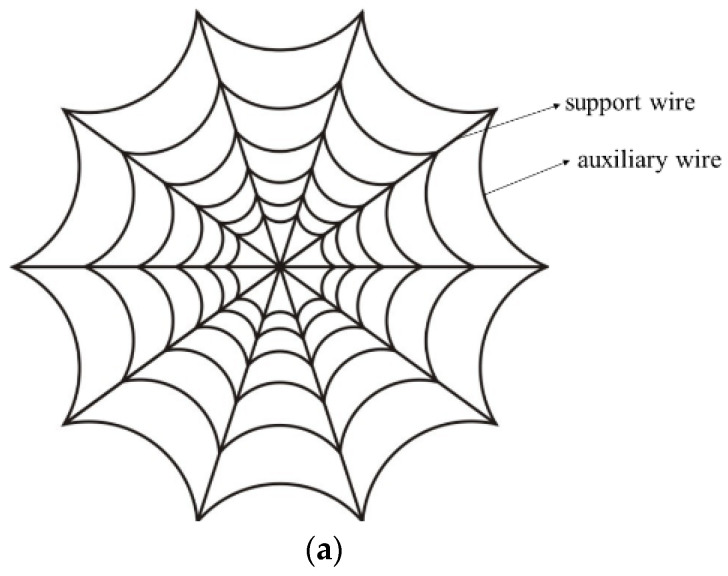

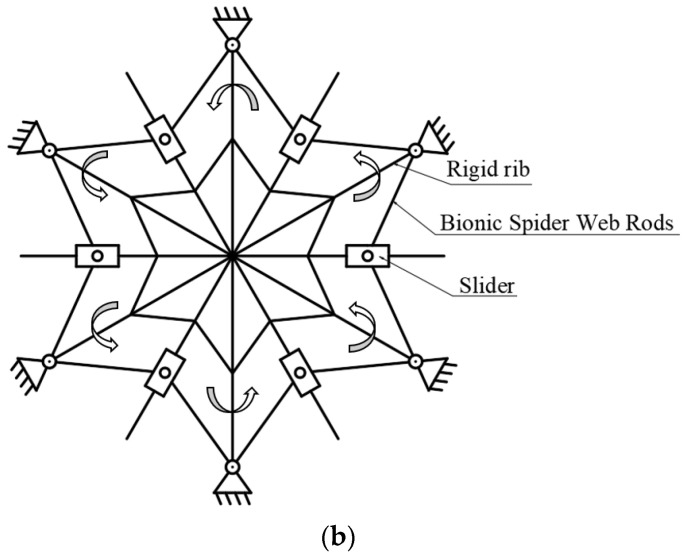
Cobweb planar structure and biomimetic evolution: (**a**) cobweb structure and (**b**) bionic evolution.

The biological structure of the chosen bionic model is initially scrutinized. In Figure 3a, the planar structure of a spider web is depicted, comprising multiple basic elements. Each element includes two support filaments and several auxiliary wires, arranged in a circumferential array. Subsequently, using the traditional rigid ribbed antenna as a reference, the spider web structure is amalgamated with the reflective surface of the space mesh antenna to facilitate bionic evolution. The evolutionary process is schematically illustrated in Figure 3b, where the active member is a rigid rib moving perpendicular to the paper surface. The conceptual evolution involves transforming two adjacent auxiliary wires on the same ring into a composite structure of two rigid members and a single slider, while the support wire evolves into a rigid rib akin to that of the traditional rigid rib antenna.

The bionic evolution structure incorporates a greater number of rigid members compared to the conventional rigid ribbed antennas. This augmentation facilitates the provision of additional mounting positions for actuators, thereby enhancing the capability for the active control of the reflective surface.

## 3. Feasibility Verification of Bionic Cobweb Scheme

**Figure 4:**
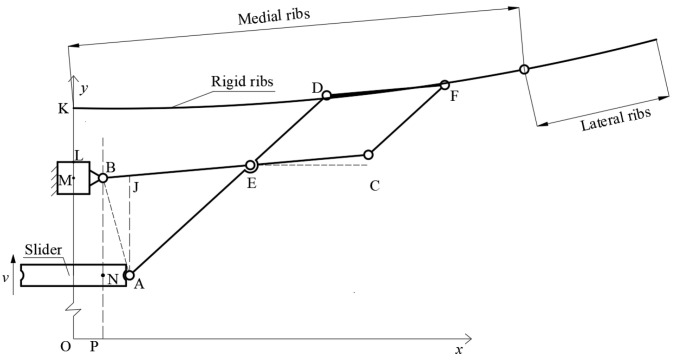
Schematic diagram of the array cell with deployable structure.

The structural foundation of the novel cable mesh design is based on a rigid ribbed antenna, featuring a reflective surface characterized by a rotating parabolic surface. The schematic representation of the fundamental unit within the expandable structure is depicted in Figure 4, where the active component is the slider. The slider undergoes a vertical movement along the y-axis, propelling the BC, AD, and CF members to rotate, ultimately resulting in the expansion of the rigid ribs. The entire expandable structure comprises 32 rigid ribs, systematically distributed in a circumferential array along the frame’s center. The design includes two sections for the rigid ribs, connected by spring hinges at the midpoint. The rib in proximity to the reflective surface’s center is designated as the inner rib, while the one that is further away is identified as the outer rib. The detailed dimensions of individual members in the expandable structure are provided in Table 1.

**Figure 5:**
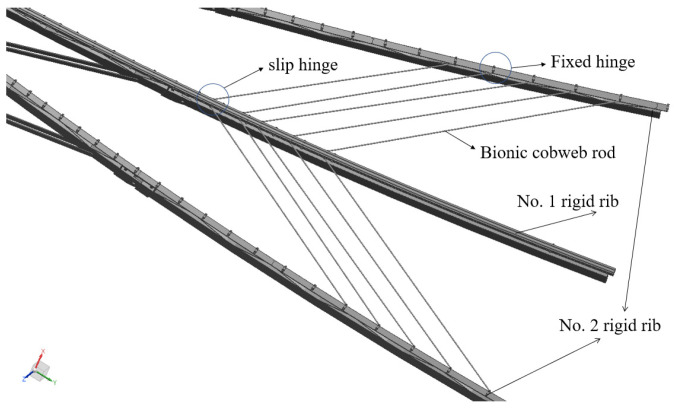
Feasibility verification model of bionic spider web scheme.

To validate the applicability of the bionic cobweb scheme in the new cable mesh design, the feasibility verification is essential. The verification model, as depicted in Figure 5, focuses on three rigid ribs, with the rigid rib housing the slip hinge designated as rigid rib No. 1, and the other two as rigid rib No. 2. Five fixed hinges are strategically positioned on the outer rib of rigid rib No. 2, away from the reflective surface’s center. An equivalent number of slip hinges are arranged on rigid rib No. 1. Fixed hinges and slip hinges are connected by bionic cobweb rigid members, with their lengths outlined in Table 2.

**Figure 6:**
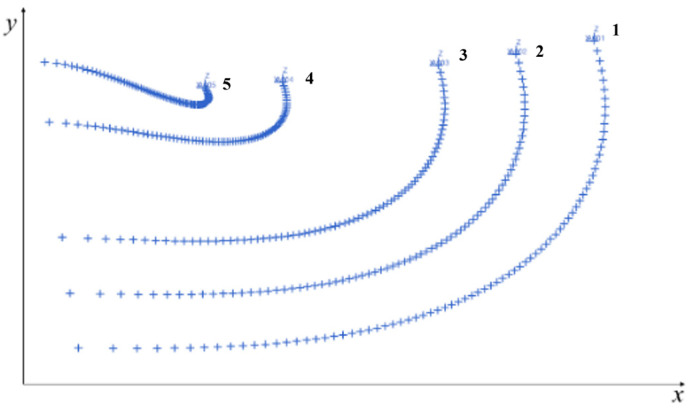
Feasibility verification results of bionic cobweb scheme (tracking trajectory of slip hinge center point).

In the UG_NX software 12.0, the trajectory of any point on the central axis of the center hinge is tracked, and the trajectory curve of each obtained tracking point is shown in Figure 6, and it can be seen that the motion of each trajectory curve shows a strong law, and there is no interruption in the movement process. Therefore, the bionic spider web scheme can be applied to the new cable mesh surface design of space mesh antennas.

## 4. Analysis of Reflective Surface Mesh Scheme of Bionic Cable Net

**Figure 7:**
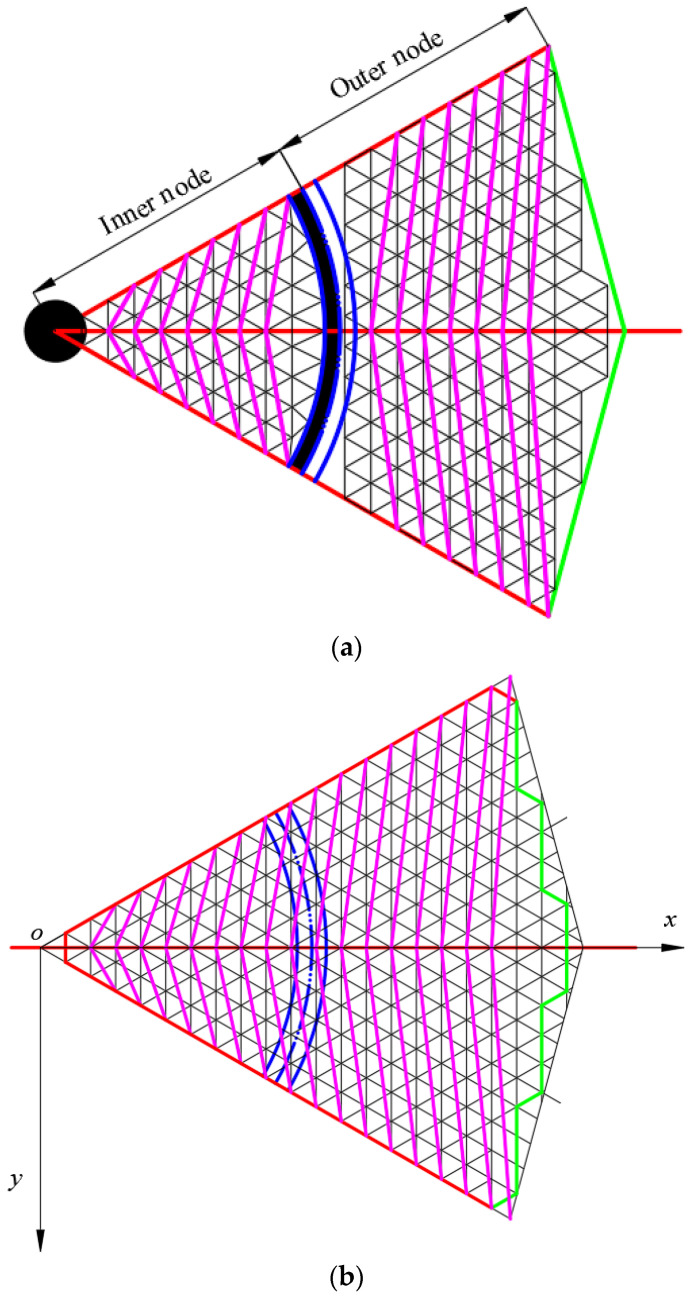

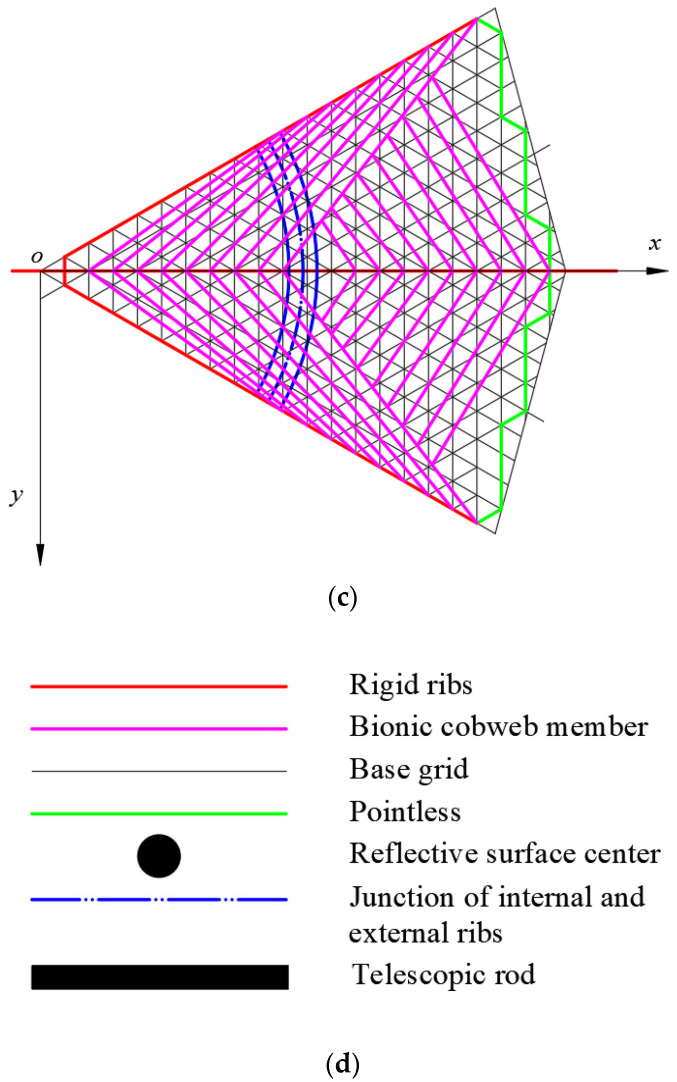
Bionic spider web plane topology design based on hexagonal meshing method: (**a**) Scheme 1, (**b**) Scheme 2, (**c**) Scheme 3, and (**d**) line interpretation.

Building upon the successful feasibility verification of the bionic spider web scheme, three meshing schemes are proposed, leveraging the existing hexagonal meshing method. These schemes are designated as follows: the “parabolic rod inside and outside independent” meshing scheme (Scheme 1, depicted in Figure 7a), the “parabolic rod inside and outside integration” meshing scheme (Scheme 2, illustrated in Figure 7b), and the “connecting rod connection-inner and outer integration” meshing scheme (Scheme 3, presented in Figure 7c). The terms “inner” and “outer” in these schemes refer to the node positions, elucidated in Figure 7d.

In Scheme 1, telescopic tie rods are introduced at the spring hinge to separate the inner and outer grids. Each segment is independently designed, with the nodes of the rigid connecting rods arranged in “inside-inside” and “outside-outside” oblique schemes.

Scheme 2 involves an integrated arrangement of rigid linkage, without the separate incorporation of telescopic tie rods. With the exception of the junction of the inner and outer ribs, which features a cross, the nodes of the rigid connecting rods elsewhere follow an “inside-inside” and “outside-outside” oblique scheme.

Scheme 3 adopts an integrated arrangement of rigid connecting rods without telescopic tie rods. The nodes of the rigid connecting rods adhere to an “outside-inside” oblique scheme. Secondary connecting rods are affixed to the outer oblique rigid connecting rod to fill the substantial mesh gap on the outside of the rigid rib. Simultaneously, a parabolic slide rail is introduced on the outer side of the middle rib, connecting the other end of the secondary connecting rod to form a secondary connecting rod system.

The analysis of the three schemes reveals the following observations:

Scheme 1: independent design inside and outside indirectly reduces the overall reflective surface’s design complexity; however, large gaps on the inner and outermost sides of the mesh may affect the overall RSA.

Scheme 2: integrated design inside and outside eliminates internal mesh gaps but increases the difficulty of controlling the accuracy of the overall reflective surface.

Scheme 3: exhibits advantages and disadvantages similar to Scheme 2, with the impact of the complex rigid connecting rod structure on the overall system stability and the RSA improvement requiring further investigation.

In conclusion, this study will comprehensively analyze and address the reflective surface accuracy for Schemes 2 and 3. The determination of the most suitable mesh topology, with the reflective surface accuracy as the evaluation criterion, will be the focus of the investigation.

Scheme 2: analysis of RSA

In this paper, the numerical integration method of axial geometric error is used to calculate the RSA, and the accuracy distribution of all nodes on the reflective surface is obtained by iterative calculation through the MATLAB R2018b software.

The calculation principle of the numerical integration method of axial geometric error is the following: if Δ*ABC* is an arbitrary triangular patch and the vertex coordinates are $A\left(x_{1},y_{1},z_{1}\right),B\left(x_{2},y_{2},z_{2}\right),C\left(x_{3},y_{3},z_{3}\right)$, then the plane equation determined by Δ*ABC* is shown in Equation (1).

(1) zs=ax+by+c

In the formula,

$$a=\frac{-y_{2}z_{1}+y_{3}z_{1}+y_{1}z_{2}-y_{3}z_{2}-y_{1}z_{3}+y_{2}z_{3}}{x_{2}y_{1}-x_{3}y_{1}-x_{1}y_{2}+x_{3}y_{2}+x_{1}y_{3}-x_{2}y_{3}}$$

$$b=\frac{x_{2}z_{1}-x_{3}z_{1}-x_{1}z_{2}+x_{3}z_{2}+x_{1}z_{3}-x_{2}z_{3}}{x_{2}y_{1}-x_{3}y_{1}-x_{1}y_{2}+x_{3}y_{2}+x_{1}y_{3}-x_{2}y_{3}}$$

$$c=\frac{x_{3}y_{2}z_{1}-x_{2}y_{3}z_{1}-x_{3}y_{1}z_{2}+x_{1}y_{3}z_{2}+x_{2}y_{1}z_{3}-x_{1}y_{2}z_{3}}{x_{2}y_{1}-x_{3}y_{1}-x_{1}y_{2}+x_{3}y_{2}+x_{1}y_{3}-x_{2}y_{3}}$$

Δ*ABC* is projected in the antenna aperture plane as Δ*A*′*B*′*C*′, and the root mean square error $\omega_{rms}$ can be obtained by dividing the area of Δ*A*′*B*′*C*′ in the aperture plane and is shown in Equation (2).

(2) $$\omega_{\mathrm{rms}}=\sqrt{\frac{1}{S_{\Delta }}\iint_{M}{\left(ax+by+c-\frac{x^{2}+y^{2}}{4f}\right)}^{2}dxdy}$$

In the formula, *M* is the points limit (Δ*A*′*B*′*C*′), and *S*_Δ_ is the area of Δ*A*′*B*′*C*′ (m^2^).

**Figure 8:**
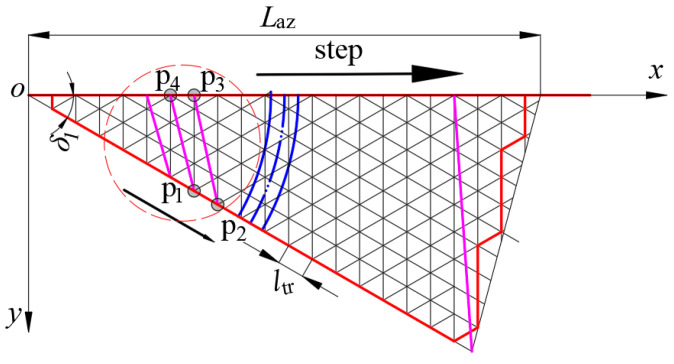
Scheme 2 mesh topology—schematic diagram of the basic elements.

In Scheme 2, the four endpoint coordinates of each independent quadrilateral membrane element coincide with the coordinates derived from the rotational parabolic equation. The structural configuration of the element is illustrated in Figure 8. The lower edge length is partitioned into u segments, with the initial base grid edge length denoted as *l*_tr_ = 20 mm. Additionally, *L*_az_ represents the length of the rigid rib projected onto the projection surface, yielding the following relationship shown in Equation (3).

(3) $$u=L_{\mathrm{az}}/l_{\mathrm{tr}}$$

To more easily represent the coordinates of each node, the given variable $x_{st}$ is shown in Equations (4) and (5).

(4) $$x_{\mathrm{st}}=l_{\mathrm{tr}}\cos(\delta_{1})$$

(5) $$\delta_{1}=2\pi /N_{g}$$

In the formula, *δ*_1_ is the angle of the projection plane of the sexual rib, and *N*_g_ is the number of rigid ribs.

The coordinates of each node in Figure 8 can be expressed in Equation (6).

(6) $$\left\{ \begin{array}{l} p_{1}:\left(ix_{\mathrm{st}},ix_{\mathrm{st}}\tan\delta_{1}\right)\\ p_{2}:((i+1)x_{\mathrm{st}},(i+1)x_{\mathrm{st}}\tan\delta_{1})\\ p_{3}:\left(ix_{\mathrm{st}},0\right)\\ p_{4}:((i-1)x_{\mathrm{st}},0) \end{array}\right.$$

The flexible rope is diagonally arranged within the quadrilateral to exert further control over the reflective surface accuracy (RSA) constituted by the base grid. The specific arrangement of the flexible rope involves an upper and lower scheme (connecting nodes 1 and 3) and a left and right scheme (connecting nodes 2 and 4).

**Figure 9:**
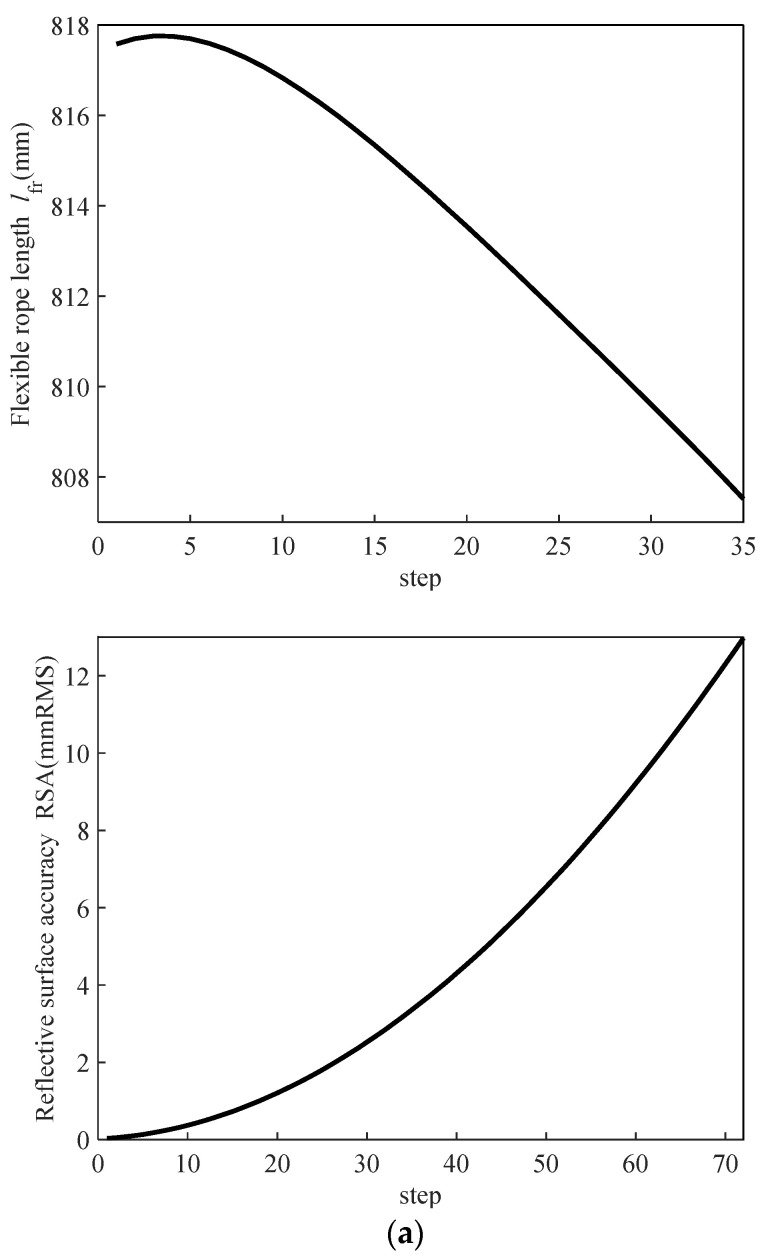

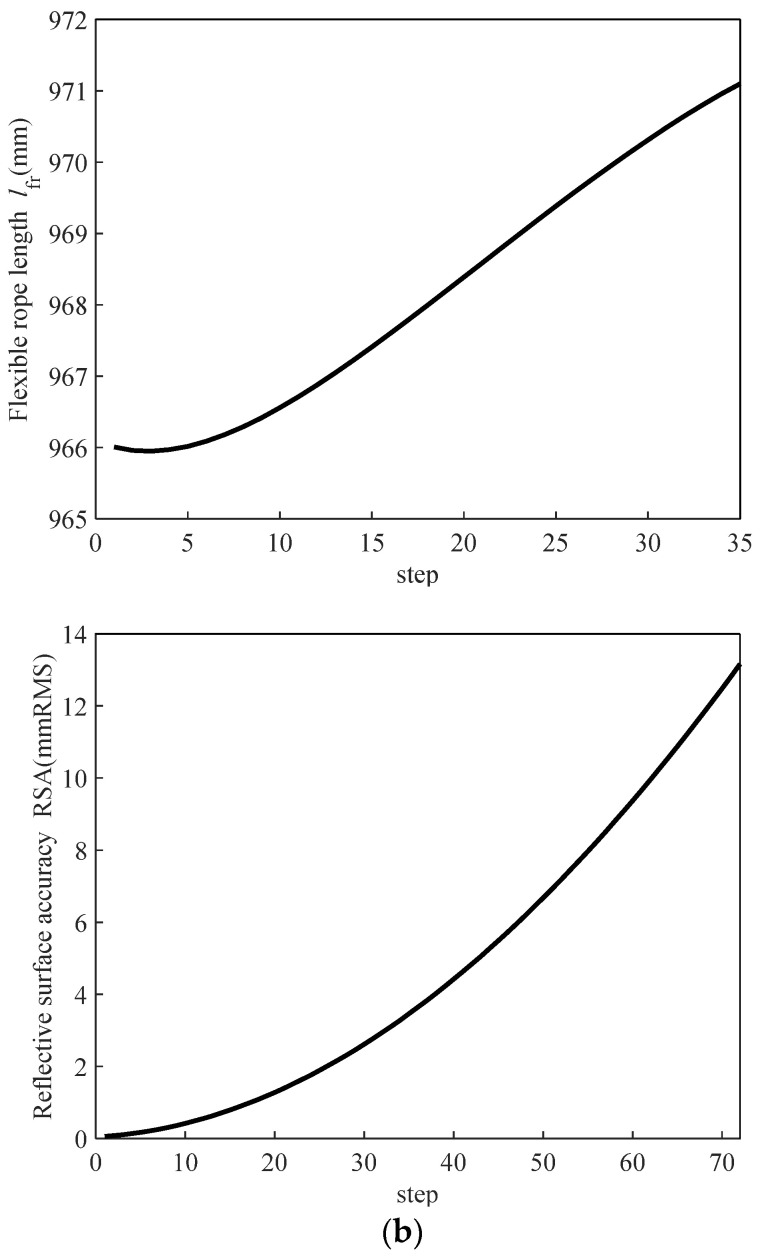
Flexible rope length and RSA variation of the basic unit of Scheme 2: (**a**) top to bottom arrangement and (**b**) left to right arrangement.

Through the derivation of motion coordinates for each node and the subsequent MATLAB calculations, the variation trends of flexible rope length and RSA during the closure of the spatial mesh antenna can be fitted. This is depicted in Figure 9, revealing that the flexible rope length in the upper and lower arrangement scheme attains its maximum value in the initial stage, while the flexible rope length in the left and right arrangement scheme reaches its minimum value during the same period. The abscissa step in Figure 9 corresponds to Figure 8, denoting the rough position of the reflective surface from the center to the outside. A step of 0 signifies that the position is at the center of the reflective surface, with larger steps indicating increasing distance from the reflective surface. The step interpretation remains consistent across the three schemes.

As the rigid rib of the antenna propels the convergence of the cable net’s reflective surface, the area of the reflective surface undergoes a transition from steady state to the maximum area and then the minimum area. Ideally, the change trend of the flexible rope should align with the area change. Any deviation could result in the decrease in the reflective surface area accompanied by the elongation of the flexible rope, leading to the risk of rope breakage. Furthermore, the analysis of the average RSA for the two flexible rope arrangement schemes (Table 3) indicates the minimal variation. In conclusion, it is determined that the upper and lower layout scheme stands as a more favorable solution.

Scheme 3: analysis of RSA

**Figure 10:**
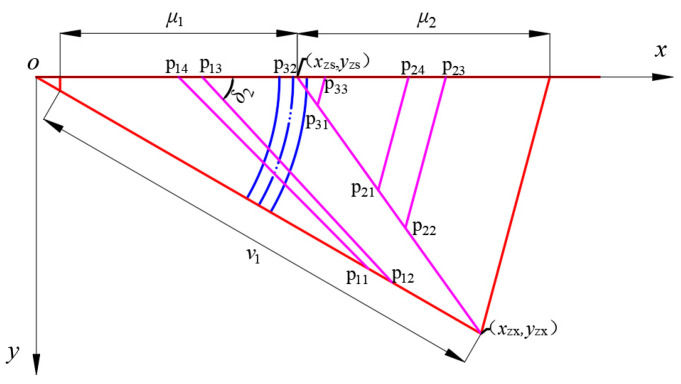
**Scheme 3** mesh topology—schematic diagram of the basic elements.

The triangular base meshing in Scenario 3 follows the same approach as Scenario 2, with the fundamental elements of the mesh plane topology illustrated in Figure 10. The membrane surface, enclosed by the coordinate origin *o*, point $\left(x_{\mathrm{zs}},y_{\mathrm{zs}}\right),$ and point $\left(x_{\mathrm{zx}},y_{\mathrm{zx}}\right)$, is referred to as the inner membrane surface. The membrane surface enclosed by points P_31_, P_32_, and P_33_ is designated as the remaining triangular membrane surface, while the membrane surface of the other remaining parts is termed the outer membrane surface. Subsequently, the terminal coordinate equations for each segment of the membrane surface will be determined individually, and the change trend of reflective surface accuracy (RSA) will be computed through the numerical integration of axial geometric errors.

Before determining the internal membranous coordinate equation, the multiplier of the upper and lower sides corresponding to *x*_st_ (that is, the number multiplied by *x*_st_) should be indicated first. The above side is the reference, and the multiplier interval of the connection point *x*_st_ is shown in Equation (7).

(7) $$\left\{ \begin{array}{l} \mu_{1}\in [3,u-\left(u-3\right)/2-1]\\ v_{1}=\mu_{1}+\left(u-3\right)/2 \end{array}\right.$$

where $\mu_{1}$ is the upper side, and $v_{1}$ is the underside.

The corresponding coordinates of each point in Figure 10 are shown in Equation (1).

(8) $$\left\{ \begin{array}{l} p_{11}:\left(v_{1}x_{\mathrm{st}},v_{1}x_{\mathrm{st}}\tan\delta_{2}\right)\\ p_{12}:((v_{1}+1)x_{\mathrm{st}},(v_{1}+1)x_{\mathrm{st}}\tan\delta_{2})\\ p_{13}:\left((\mu_{1}+1)x_{\mathrm{st}},0\right)\\ p_{14}:(\mu_{1}x_{\mathrm{st}},0) \end{array}\right.$$

In this scheme, the lower nodes of the outer membrane surface are all placed on the rightmost rigid member of the inner membrane surface, in order to accurately represent the endpoint coordinates of the outer membrane surface, and the *x* coordinate of the end rigid member is shown in Equation (9).

(9) $$\left\{ \begin{array}{l} x_{\mathrm{zs}}:\left(\left(u-\frac{u-3}{2}\right)x_{\mathrm{st}},0\right)\\ x_{\mathrm{zx}}:\left(ux_{\mathrm{st}},ux_{\mathrm{st}}\tan\delta_{2}\right) \end{array}\right.$$

The number of aliquots $N_{e}$ of the end rigid member is determined as shown in Equation (10).

(10) $$N_{e}=\left\lfloor \frac{L_{\mathrm{az}}}{x_{\mathrm{st}}}\right\rfloor -\left(u-\frac{u-3}{2}\right)$$

The equation of the coordinates of each node of the outer membrane surface is shown in Equation (11).

(11) $$\left\{ \begin{array}{l} p_{21}:\left(x_{\mathrm{zs}}+\frac{x_{\mathrm{zx}}-x_{\mathrm{zs}}}{N_{e}}\mu_{2},y_{\mathrm{zs}}+\frac{y_{\mathrm{zx}}-y_{\mathrm{zs}}}{N_{e}}\mu_{2}\right)\\ p_{22}:\left(x_{\mathrm{zs}}+\frac{x_{\mathrm{zx}}-x_{\mathrm{zs}}}{N_{e}}\left(\mu_{2}+1\right),y_{\mathrm{zs}}+\frac{y_{\mathrm{zx}}-y_{\mathrm{zs}}}{N_{e}}\left(\mu_{2}+1\right)\right)\\ p_{23}:\left(\left(\mu_{2}+u-\frac{u-3}{2}+1\right)x_{\mathrm{st}},0\right)\\ p_{24}:\left(\left(\mu_{2}+u-\frac{u-3}{2}\right)x_{\mathrm{st}},0\right) \end{array}\right.$$

where $\mu_{2}\in \left[1,N_{e}-1\right]$.

The coordinate equation for the remaining triangular membrane face can be expressed in a similar way to the outer membrane face and is shown in Equation (12).

(12) $$\left\{ \begin{array}{l} p_{31}:\left(x_{\mathrm{zs}}+\frac{x_{\mathrm{zx}}-x_{\mathrm{zs}}}{N_{e}},y_{\mathrm{zs}}+\frac{y_{\mathrm{zx}}-y_{\mathrm{zs}}}{N_{e}}\right)\\ p_{32}:\left(\left(u-\frac{u-3}{2}\right)x_{\mathrm{st}},0\right)\\ p_{33}:\left(\left(u-\frac{u-3}{2}+1\right)x_{\mathrm{st}},0\right) \end{array}\right.$$

**Figure 11:**
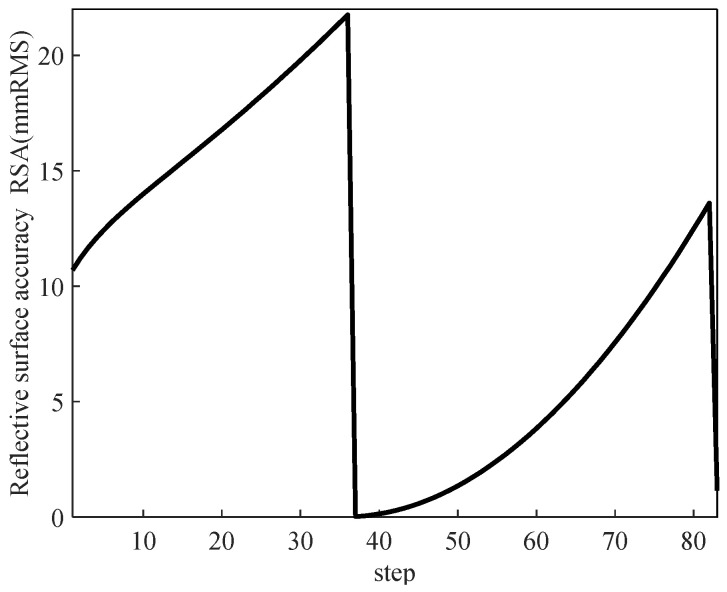
Variation trend of reflective surface accuracy in Scheme 3.

The overall RSA calculation of Scheme 2 was completed in MATLAB, and the change trend was obtained as shown in Figure 11, which shows that compared with Scheme 2, the maximum RSA of the internal film surface of Scheme 3 increased, and the overall value was large, which was very unfavorable to the later improvement; its average RSA was 9.77 mmRMS, which was double compared to that of Scheme 2.

In summary, the final scheme is determined as a scheme of up–down arrangement (nodes 1 and 3 connected) and flexible rope in Scheme 2 (“parabolic rod inner and outer integration” meshing scheme).

## 5. Bionic Reflective Surface Structural Design

The balanced rigid connecting rod design considers *l*_tr_ = 30 mm, and *L*_az_ = 1500 mm, yielding the theoretical average RSA = 4.8187 mmRMS, the maximum RSA = 13.3644 mmRMS, and the minimum RSA = 0.0748 mmRMS. Upon solving for RSA, it is observed that when the antenna diameter exceeds 1560 mm, RSA deteriorates significantly, compromising the reflective surface’s accuracy, making it challenging to meet the actual requirements of the space antenna. Therefore, to align with the practical needs of the space mesh antenna and considering the existing active control scheme, the antenna diameter is adjusted to 1500 mm, and the modified reflective surface is pre-designed.

**Figure 12:**
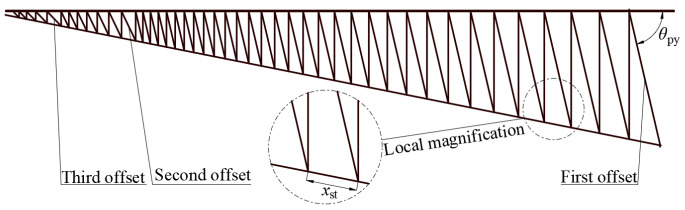
Bionic cable net reflective surface mesh design.

Upon determining the length of each bionic cobweb rigid member, the motion simulation in UG reveals a motion error starting from the 15th set of members. Further analysis attributes the incomplete motion to the sliding hinge of the member reaching its limit during the transition of the antenna reflective surface from the final state to the maximum area morphology. This limitation occurs due to the insufficient length of the bionic cobweb rigid member or an overly small offset angle of the projection surface (Figure 12) of the initial rigid member.

Key conditions for the design of bionic cobweb rigid members include ensuring that the offset angle of each set of members allows the sliding hinge on the sleeve members to move outward, preventing insufficiency in the length of the bionic cobweb rigid members.

After establishing the crucial conditions for rigid member design, the mesh is created in UG_NX, as depicted in Figure 12. The actual rigid member length is determined through curve projection, and kinematics simulation is validated. Figure 12 displays a total of 44 rigid members on the bionic cable mesh, labeled from the outside to the center as the first set to the forty-fourth set. The length of the initial member (the first set of members) is set to 575.5 mm, the three offset angles are 76.9°, 64.4°, and 46.1°, and the initial lower spacing is set to $x_{st}\geq 30\;mm$.

Motion simulation was conducted for sets 1–2, 7–8, 13–16, 21–26, 31–32, 37–38, and 43–44 of rigid rod sets. However, it was observed that the entire simulation process could not be completed starting from the 27th set of rigid rods. The measured fixed hinge projection spacing for sets 25–26 and 26–27 of rigid rod sets is 42.7 mm and 40.7 mm, respectively. Consequently, the minimum fixed hinge point spacing was set to *x*_st_ = 42.7 mm, with adjustments made to the offset angle when the fixed hinge point spacing is *x*_st_ < 42.7 mm. Following several iterations, the obtained offset angles for each set of bionic cobweb rigid rod sets are presented in Table 4.

## 6. Design and Accuracy Analysis of the Overall Reflective Surface of the Spatial Mesh Antenna

**Figure 13:**
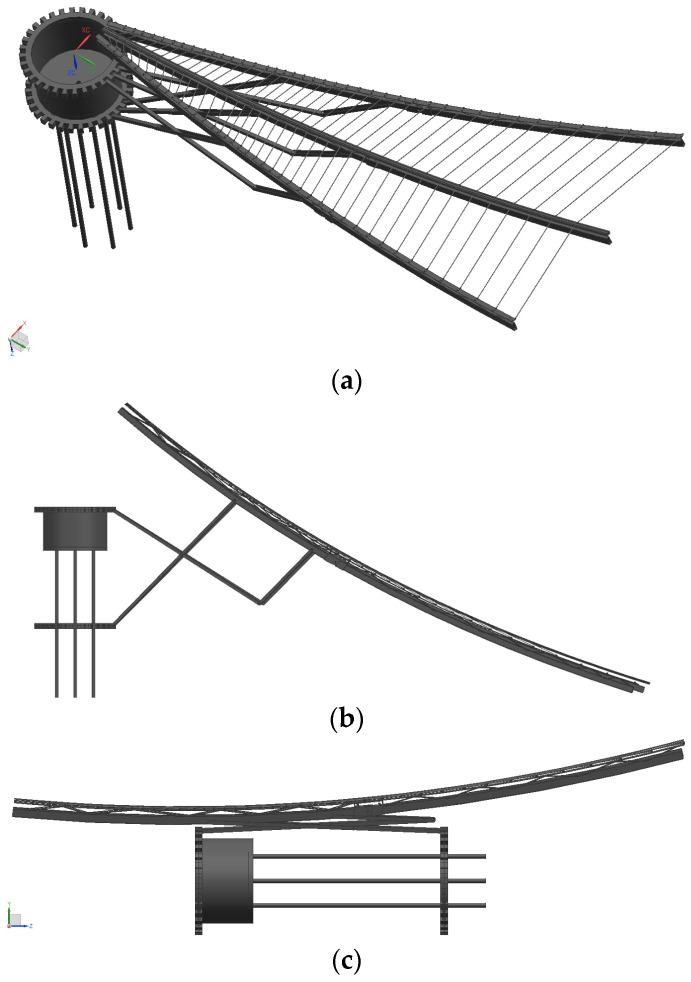
Various forms of cable net antenna: (**a**) unfolded state, (**b**) semi-gathered state, and (**c**) gathered state.

The three-dimensional modeling of the designed bionic cable mesh surface has been executed, and the motion simulation has been completed in the software. The unfolded form, semi-converged state, and convergence form of the cable mesh surface are illustrated in Figure 13. At this stage, the reflective surface of the spatial mesh sky cable net comprises only 44 sets of rigid members distributed in a circle. The grid area divided by each member is large, making it challenging to ensure the reflective surface accuracy (RSA) on such a rigid structure. Additionally, there is no available position to arrange the actuator. Hence, the further refinement of the flexible woven mesh layout is necessary.

**Figure 14:**
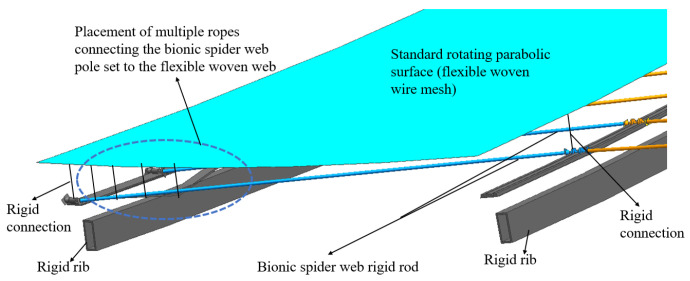
The layout scheme of the flexible woven net.

Focusing on the outermost quadrilateral cable network unit, the tension cable network is arranged according to the scheme shown in Figure 14, and a comprehensive accuracy analysis of the entire quadrilateral cable network element is conducted. In Figure 14, the flexible cable net system is positioned between the rigid members of the bionic cobweb and the flexible woven web. The two sides of the bionic cobweb rigid rod group are rigidly connected and fixed, while the middle part features a flexible connection. The bionic spider web rigid rod group and the flexible woven net are connected by a series of short ropes, and controlling the length of these short ropes can bring the reflective surface closer to the standard rotating parabolic surface.

**Figure 15:**
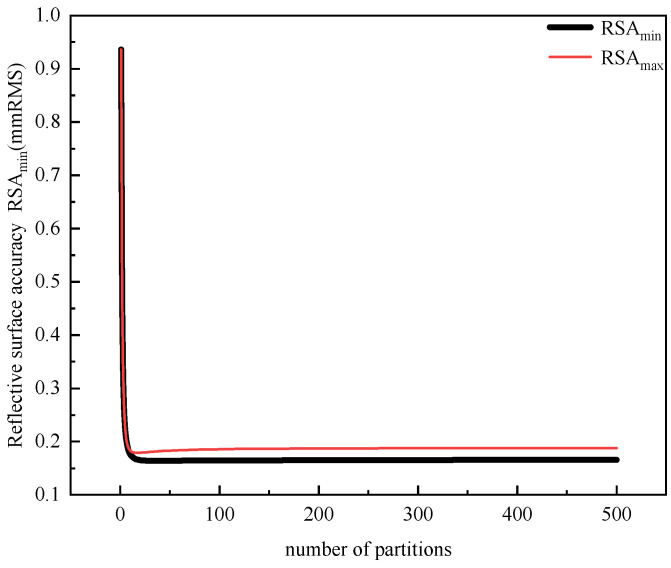
The variation trend of RSA under different subdivisions.

Preliminary research indicates that the number of nodes (i.e., the number of short ropes in the flexible connection part) influences the RSA. Figure 15 depicts the change trend of the maximum RSA and the minimum RSA corresponding to node numbers ranging from 0 to 500. It is observed that when the number of nodes exceeds 26, the change in RSA becomes negligible. The number of nodes is further defined as the number of subdivisions of the long side (i.e., the longest side in each quadrilateral membrane element). The ratio of the longest bionic spider web member to the maximum number of nodes is defined as the minimum subdivision length.

Two flexible woven mesh layout schemes are proposed and are follows:

(1) Flexible woven web layout scheme with a fixed subdivision number (layout plan 1): each bionic spider web member adopts the same number of subdivisions.

(2) Flexible woven web layout scheme with a variable subdivision number (layout plan 1): each bionic cobweb member adopts a different number of subdivisions, ensuring that the subdivision length of each bionic cobweb member does not exceed the upward integer value of the minimum subdivision length.

**Figure 16:**
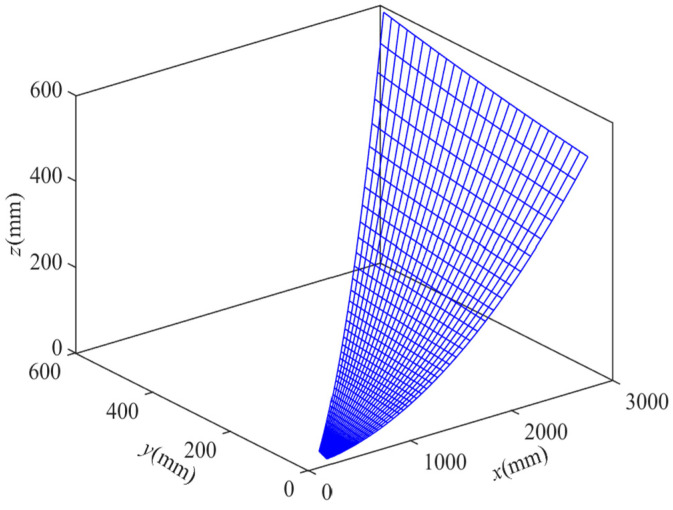
Mesh design of layout plan 1.

**Figure 17:**
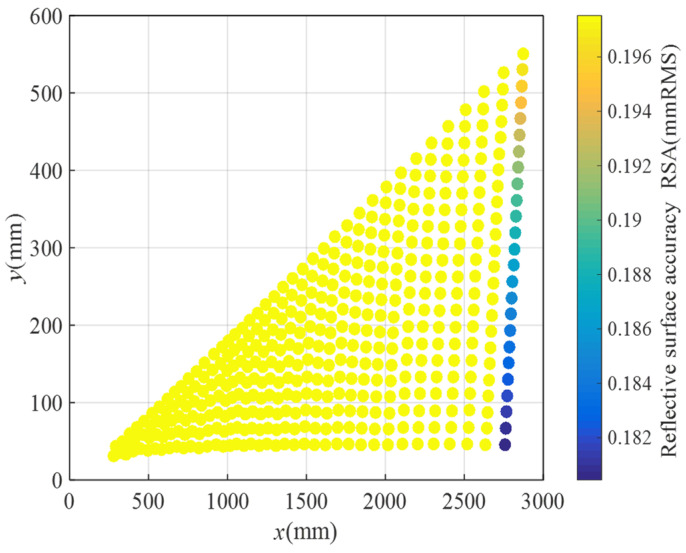
RSA distribution for layout plan 1.

The grid design of the flexible woven mesh layout scheme with a fixed number of subdivisions is illustrated in Figure 16, and the distribution of RSA obtained by the solution is presented in Figure 17.

Before solving the RSA of the flexible woven web arrangement scheme with variable subdivision number, the length ${l_{f}}_{i}$ of all bionic spider web members should first be obtained (Table 5), and then, the number of subdivisions of each member $s_{fi}$ should be determined according to the set range. The length of the bionic cobweb member and the number of subdivisions should be satisfied:

(13) $$\varepsilon_{fi}=l_{fi}/s_{fi}\leq \left\lceil \varepsilon_{f1}\right\rceil$$

where ${\varepsilon_{f}}_{i}$ is the minimum subdivision length. If there are multiple $s_{fi}$ that satisfy the condition at the same time, the smallest of all $s_{fi}$ that meets the condition should be taken. The final subdivision of all bionic cobweb members is shown in Table 6.

**Figure 18:**
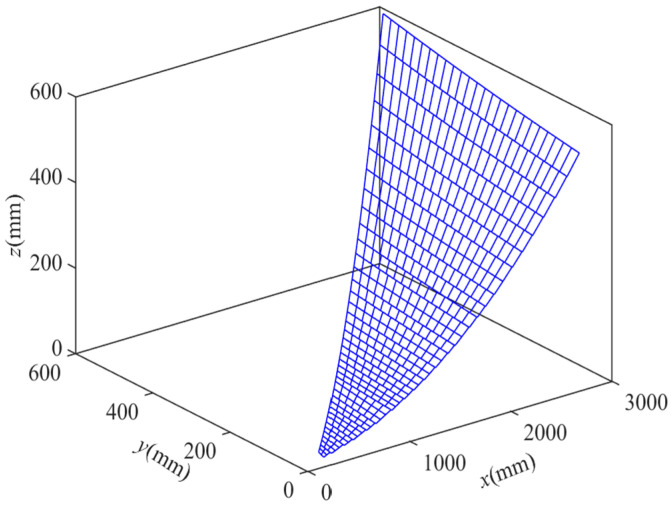
Mesh design of layout plan 2.

**Figure 19:**
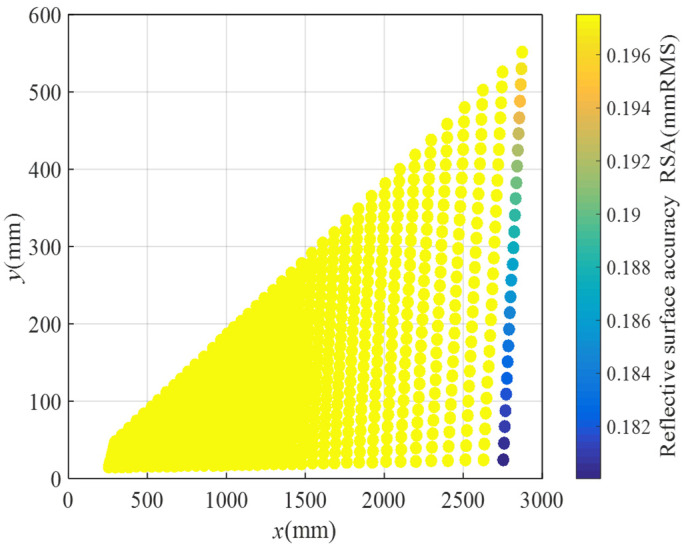
RSA distribution for layout plan 2.

The coordinates of all nodes have been computed using MATLAB, and the grid design for the flexible woven net layout scheme with variable subdivision numbers is depicted in Figure 18. Following a sequence of node solving, connecting, and drawing steps, it was observed that, since the subdivision numbers of different bionic cobweb members are not precisely the same, two subdivisions on the same bionic cobweb member are necessary simultaneously, indirectly achieving a further division. The reflective surface accuracy (RSA) of the scheme is determined using MATLAB, and the results are presented in Figure 19. It is evident that, except for a very small part of the cable mesh surface, the RSA is less than 0.196 mmRMS, with 95% of the RSA measuring 0.196 mmRMS.

Upon comparison, it is noted that the difference in RSA obtained by the two schemes is minimal. Therefore, considering economic factors, the flexible woven mesh layout scheme with a variable subdivision number is selected. At this point, the configuration design of the reflective surface of the cable mesh is concluded, and the RSA of the cable net is attained through optimization and step-by-step screening. The final cable mesh achieves an RSA of 0.196 mmRMS.

## Figures and Tables

**Table 1:** The spatial mesh antenna deploys the individual member lengths of the structure (unit: mm).

*L*_BN_	*L*_PB_	*L*_AE_	*L*_BE_, *L*_EC_, and *L*_DF_	*L*_DE_	*L*_KL_
4.2	518.2	268.53	250	231.47	73.41

**Table 2:** The bionic spider web scheme validates the length of individual members in the model.

**No.**	1	2	3	4	5
**Length/mm**	904	871	838	778	751

**Table 3:** Average RSA of different flexible rope arrangement schemes.

**Scheme**	1 and 3 connection	2 and 4 connection
**Average RSA**	4.8128 mmRMS	4.7222 mmRMS

**Table 4:** Cable net reflective surface design—offset solution result.

**No.**	1–26	27–32	33–35	36–37	38–39	40	41
***θ*_py_/°**	76.9	70	63	56	49	42	38

**Table 5:** The length of each bionic cobweb rigid member.

NO.	Length (mm)	NO.	Length (mm)	NO.	Length (mm)	NO.	Length (mm)
1	579.968676	12	342.9581088	23	203.4144425	34	105.3328486
2	552.82172	13	327.0255712	24	193.994366	35	94.64464945
3	526.9482283	14	311.839783	25	185.0119595	36	91.42073962
4	502.3123383	15	297.3649534	26	176.4467864	37	79.14215885
5	478.8512538	16	283.5669513	27	174.5405131	38	75.27396839
6	456.506137	17	270.413561	28	161.845411	39	62.24837076
7	435.221317	18	257.8741173	29	150.1051991	40	58.06776318
8	414.9441491	19	245.9194149	30	139.219381	41	49.16385111
9	395.6249944	20	234.5217674	31	129.1251502		
10	377.216852	21	223.6549038	32	119.7647449		
11	359.6751978	22	213.2937715	33	117.1965809		

**Table 6:** The number of subdivisions of each cobweb-like rigid member.

Set	Number of Segments	Set	Number of Segments	Set	Number of Segments	Set	Number of Segments
1	26	7~8	19	15~16	13	29~30	7
2	25	9	18	17~18	12	31~33	6
3	23	10	17	19~20	11	34~35	5
4	22	11	16	21~22	10	36~38	4
5	21	12~13	15	23~25	9	39~41	3
6	20	14	14	26~28	8		

## Data Availability

Data are contained within the article.
